# The predictive value of neutrophil-to-lymphocyte ratio for the occurrence, progression, and mortality of diabetic nephropathy: a systematic review and meta-analysis

**DOI:** 10.1038/s41598-025-30680-4

**Published:** 2026-01-05

**Authors:** Hao Liu, Lvlin Chen, Junchen Zhu, Zhen Zhang, Haiyan Nie, Chao Tan, Wang Gong, Yinquan Ai, Xiang Yuan, Sen Zhang, Xingyu He, Xinyu Liu, Peiling Cai, Yongyan Song

**Affiliations:** 1 Clinical Medical College & Affiliated Hospital & College of Basic Medicine, Chengdu University, Chengdu, 610081 Sichuan China; 2 Department of Critical Care Medicine, Affiliated Hospital of Chengdu University, Chengdu, 610081 China

**Keywords:** Neutrophil-to-lymphocyte ratio, Diabetic nephropathy, Diabetes mellitus, Kidney function deterioration, Mortality, Meta-analysis, Biomarkers, Diseases, Endocrinology, Medical research, Nephrology, Risk factors

## Abstract

The neutrophil-to-lymphocyte ratio (NLR) has emerged as a promising inflammatory biomarker in diabetic nephropathy (DN). However, its predictive value across the clinical spectrum of DN, from occurrence to progression and mortality, remains undefined. This study aimed to systematically evaluate the association between NLR and the risk, progression, and mortality of DN through a comprehensive meta-analysis. This systematic review and meta-analysis searched six databases for cohort and case-control studies published up to May 28, 2025. Pooled odds ratios (ORs) and standardized mean differences (SMDs) were calculated. Subgroup and sensitivity analyses explored heterogeneity. This review was prospectively registered in PROSPERO (CRD42024586927). Thirty-nine studies involving 14,300 participants showed that patients with diabetic nephropathy (DN) had higher NLR levels than diabetic controls without nephropathy, demonstrating a significant association between elevated NLR and DN occurrence (SMD = 1.31, 95% CI 0.96–1.66; OR = 2.16, 95% CI 1.85–2.52; both *P* < 0.00001). Among DN patients, those who experienced kidney function deterioration also exhibited higher NLR values than those with stable kidney function (SMD = 1.02, 95% CI 0.77–1.26, *P* < 0.00001; OR = 2.12, 95% CI 1.04–4.31, *P* = 0.04). The association with mortality was marginally non-significant (OR = 1.21, 95% CI 0.99–1.48, *P* = 0.06). Subgroup analyses showed stronger associations in patients aged ≥ 60 years and those with BMI ≥ 25 kg/m². NLR shows potential as a biomarker for identifying individuals at increased risk of DN occurrence and progression. Given the heterogeneity and possible publication bias among studies, these findings should be interpreted with caution and confirmed by future large-scale, standardized research.

**Supplementary Information:**

The online version contains supplementary material available at 10.1038/s41598-025-30680-4.

## Introduction

Diabetes mellitus (DM) is a globally prevalent endocrine disorder that presents a substantial health burden^1,2^. Without timely intervention, patients with DM often develop a range of complications, including diabetic nephropathy (DN), diabetic retinopathy, diabetic neuropathy, and atherosclerotic cardiovascular and cerebrovascular conditions^3–6^. DN is one of the most prevalent and severe microvascular complications of DM, often resulting from sustained hyperglycemia^7^. According to current KDIGO, ADA, and AACE guidelines, the term diabetic kidney disease (DKD) is preferred to describe clinically diagnosed kidney involvement in diabetes. In this study, the term DN is used to reflect the terminology adopted in the included studies, all of which relied on clinical rather than biopsy-based diagnostic criteria. Its pathogenesis involves chronic inflammation, oxidative stress, tubulointerstitial fibrosis, and hemodynamic alterations in kidney blood flow^8–11^. The early manifestation of DN may include microalbuminuria. However, as the disease progresses, it may develop into overt proteinuria and kidney function deterioration, eventually leading to end-stage kidney disease and increasing the risk of mortality. Current diagnostic methods for DN, such as urine albumin-to-creatinine ratio (UACR) and estimated glomerular filtration rate (eGFR), are influenced by factors like posture, exercise, infections, and dehydration, which may lead to false-positive or false-negative results^12^. Additionally, kidney biopsy and imaging techniques, though effective, are invasive, costly, and carry certain risks, limiting their routine use for early diagnosis^13,14^. Thus, establishing a simple and effective clinical diagnostic approach for DN remains a pressing need^15–18^.

Advancements in medical testing technologies have positioned the neutrophil-to-lymphocyte ratio (NLR) as an easily obtainable and cost-effective marker of inflammation^19^. Neutrophils, as central components of the innate immune system, play a crucial role in host defense against acute infections, particularly those of bacterial and fungal origin, by eliminating pathogens through phagocytosis^20,21^. Lymphocytes, including T cells, B cells, and natural killer cells, are essential for mediating humoral and cellular immune responses, sustaining immune memory, and performing immune surveillance^22,23^. The NLR offers a simple and rapid means to assess both immune function and the extent of systemic inflammation^24,25^. An elevated NLR is often reflective of chronic low-grade inflammation, a known contributor to the pathogenesis and progression of DN^26^. Increased neutrophil activation and lymphocyte suppression in diabetes can amplify oxidative stress, endothelial injury, and kidney fibrosis, thereby linking systemic inflammation to structural and functional kidney decline. Recent studies have demonstrated associations between NLR and both the progression and prognosis of DN^27–34^. For instance, Liu et al.^34^ reported an association between NLR and the occurrence of DN as well as kidney function deterioration. However, contrary to other findings, Cao et al.^35^ conducted a case-control study and reported no significant association between NLR and kidney function deterioration in DN, highlighting the need for further systematic evaluation of its predictive utility. Therefore, whether NLR can be used as a marker for diagnosis and prognosis in DN still needs to be clarified.

While previous meta-analyses have partially examined the relationship between NLR and DN, most have concentrated on the early stages of disease occurrence, without assessing longitudinal progression or key clinical outcomes such as kidney function deterioration and mortality^36,37^. Furthermore, the absence of subgroup analyses in earlier studies has limited the identification of population-specific patterns and potential risk modifiers. In this context, the present study systematically traces the natural course of DN from initial onset through progression to adverse outcomes, comprehensively evaluating the predictive value of NLR across the entire clinical spectrum. By incorporating both continuous and categorical NLR measurements, and conducting subgroup analyses stratified by age, BMI, glycemic control, and geographic region, the study delivers a nuanced and clinically relevant assessment of NLR in DN risk stratification and management. These methodological advances provide robust and actionable evidence, filling critical gaps in current DN risk prediction strategies and supporting the integration of NLR into precision medicine approaches. Specifically, this study aimed to quantify the association between NLR and DN occurrence, assess its predictive value for kidney function deterioration, and evaluate its association with all-cause mortality.

## Materials and methods

### Literature search

This study strictly followed the Preferred Reporting Items for Systematic Reviews and Meta-Analyses (PRISMA 2020) guidelines^38^, and its protocol was formally registered in the International Prospective Register of Systematic Reviews (PROSPERO: CRD42024586927). The search strategy was collaboratively designed by 2 investigators, LH and ZZ, who independently constructed subject terms and keywords to perform a comprehensive search of multiple databases, including PubMed, Embase, Web of Science, Cochrane Library, CNKI, and Wanfang, covering the period from the inception of each database to May 28, 2025. In response to reviewer comments during the revision stage, CLL and ZJC supplemented additional search terms to enhance the comprehensiveness of the literature retrieval, and further extended the search period to October 28, 2025, building on the initial search strategy designed by LH and ZZ. The search encompassed a broad spectrum of terms, including “diabetic nephropathies” “nephropathies, diabetic” “nephropathy, diabetic” “diabetic nephropathy” “neutrophils” “lymphocytes” and “neutrophil-to-lymphocyte ratio, NLR”.

### Study selection

We included studies according to the PICOS principle (Population, Comparison, Outcome, and Study Design; the “Intervention” component was not applicable because all included studies were observational in nature):

Occurrence of DN: P: diabetic population; C: participants with DN versus those without DN; O: NLR levels (as a continuous or categorical variable); S: cohort or case-control studies. DN occurrence was diagnosed according to the KDIGO diagnostic criteria^39^.

Clinical outcomes of DN: P: DN population; C: participants with high versus low NLR; O: kidney function deterioration and mortality; S: cohort or case-control studies.

Kidney function deterioration was defined in accordance with KDIGO criteria^40^ and the original study settings, including: (1) a decline in eGFR from 60 to 90 to < 60 ml/min/1.73m^2^, or (2) an increase in UACR from 30 to 300 to > 300 mg/g. To ensure comprehensive inclusion, studies reporting other forms of overt kidney impairment (e.g., dialysis initiation, or significant Scr elevation) were also included and classified as “others.” To mitigate heterogeneity, subgroup analyses by diagnostic marker (eGFR, UACR, others) were performed (Table 3).

**Fig. 1:**
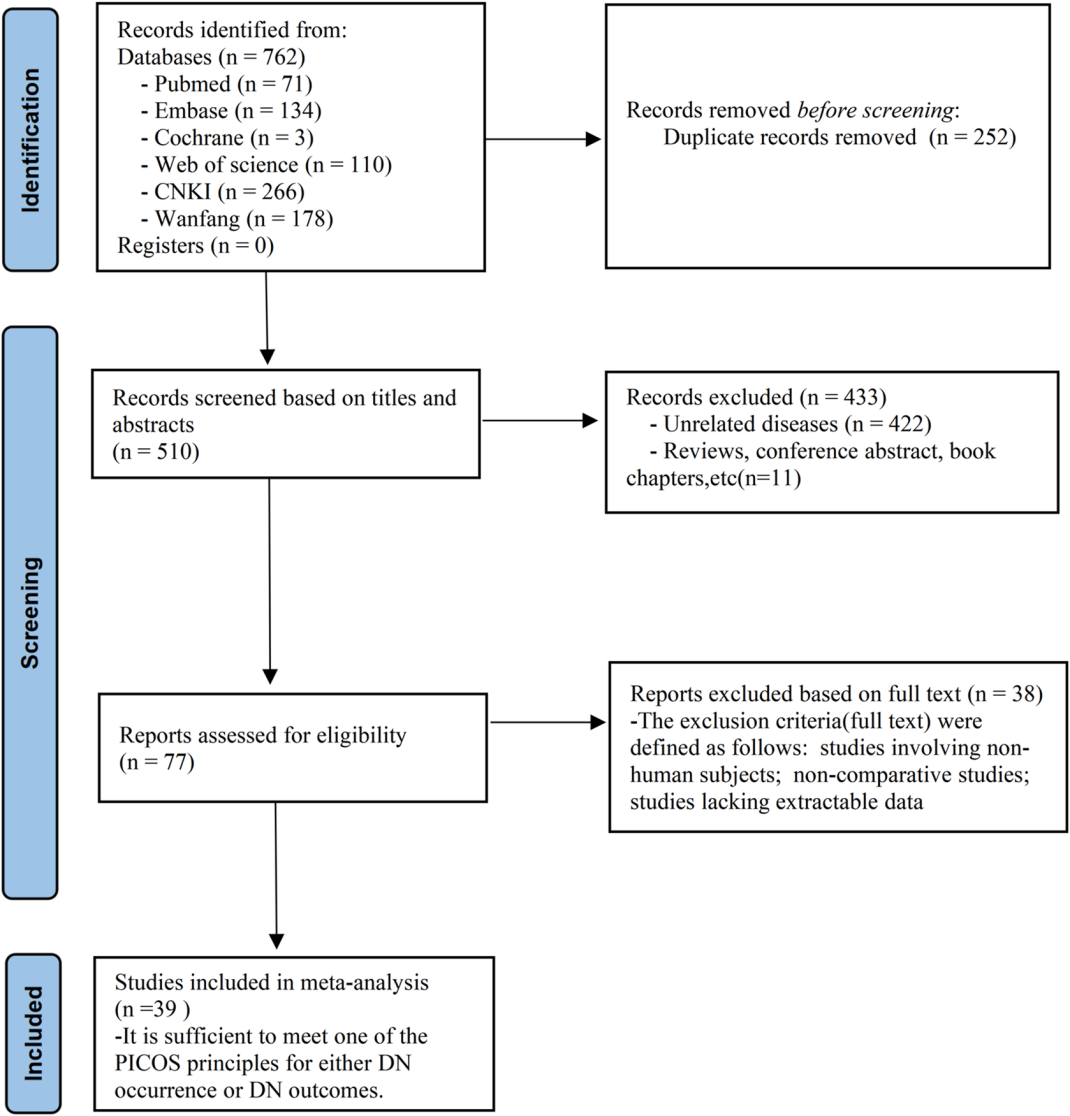
Flow chart of literature screening.

A unified process using both PICOS frameworks simultaneously was employed. Studies meeting either of the criteria were included, and subsequently, data extraction was divided into two analytic categories: (a) DN occurrence, and (b) DN clinical outcomes (kidney function deterioration and mortality). This process is illustrated in Fig. 1.

The exclusion criteria were defined as follows: (1) studies involving non-human subjects; (2) non-comparative studies; (3) studies not addressing DN; (4) studies lacking extractable data; (5) non-original articles, such as letters, reviews, and editorials.

Two researchers, LH and ZZ, independently reviewed the titles and abstracts of all retrieved records, evaluated the full texts, and determined eligibility according to the inclusion criteria, with any disagreements resolved by mutual consensus. During the revision stage, to address reviewer comments and ensure comprehensiveness, CLL and ZJC further reviewed the newly retrieved articles and excluded ineligible studies, with their screening results cross-validated with the original research team.

### Data extraction

Researchers NHY and TC independently carried out the initial data extraction process. CLL and ZJC further re-extracted and verified study-level data to ensure accuracy, in line with the revised analytical requirements. Any discrepancies were subsequently resolved through consensus among all contributing authors. The extracted data encompassed a variety of parameters: the first author’s name, year of publication, country of the study, study design, sample size, patient demographics (age and gender), body mass index (BMI), fasting plasma glucose (FPG), eGFR, blood pressure, hemoglobin A1c (HbA1c), duration of diabetes, UACR, serum creatinine, cutoff values for study measures, NLR, occurrence of DN, and clinical outcomes of DN, including kidney function deterioration, and mortality. Cutoff values for NLR were obtained directly from the original studies, which determined optimal thresholds using receiver operating characteristic (ROC) curve analysis. These cutoffs were not recalculated in our study. Variability in cutoff definitions across studies was addressed through subgroup analyses stratified by NLR cutoff levels (< 2.4 vs. ≥2.4), as presented in Tables 1 and 2, to account for potential heterogeneity arising from different threshold settings.

**Table 1:** Meta-analyses between NLR as a continuous variable and DN risk.

Groups/subgroups	*N*	SMD [95%CI]	*P* value	I^2^(%)
Total	19	1.31 [0.96–1.66]	< 0.00001	95
Region				
South Asia	8	1.43 [0.86–2.01]	< 0.00001	95
East Asia	5	0.79 [0.33–1.26]	0.0009	94
West Asia	4	1.25 [0.41–2.10]	0.003	94
Mean/median age				
≥ 60	4	0.92 [0.59–1.24]	< 0.00001	75
< 60	15	1.42 [0.96–1.88]	< 0.00001	96
NLR cutoff				
≥ 2.4	3	0.81 [0.42–1.20]	< 0.0001	80
< 2.4	6	1.54 [0.80–2.29]	< 0.0001	95
BMI				
≥ 25	9	1.24 [0.79–1.70]	< 0.00001	94
< 25	6	1.61 [0.76–2.46]	0.0002	98
FPG				
≥ 9	5	1.44 [0.41, 2.46]	0.006	97
< 9	3	1.12 [0.70, 1.55]	< 0.00001	87
Diagnostic markers				
UACR and equivalents	15	1.47 [1.07, 1.88]	< 0.00001	95
eGFR	4	0.71 [0.14, 1.28]	0.02	93

NLR, neutrophil-to-lymphocyte ratio; DN, diabetic nephropathy; SMD, standardized mean difference; CI, confidence interval; BMI, body mass index; FPG, fasting plasma glucose; eGFR, estimated glomerular filtration rate; UACR, urinary albumin-to-creatinine ratio.

**Table 2:** Meta-analyses between NLR as a categorical variable and DN risk.

Groups/subgroups	*N*	OR [95%CI]	*P* value	I^2^ (%)
Total	14	2.16 [1.85–2.52]	< 0.00001	43
Study design				
Cohort	6	1.85 [1.53–2.25]	< 0.00001	22
Case-control	8	2.36 [1.96–2.84]	< 0.00001	29
Region				
China	10	2.08 [1.76–2.45]	< 0.00001	49
Japan	3	2.84 [1.26–6.38]	0.01	29
Mean/median age				
≥ 60	8	2.29 [1.84–2.86]	< 0.00001	58
< 60	6	1.94 [1.58–2.37]	< 0.00001	3
NLR cutoff				
≥ 2.4	3	2.30 [1.50, 3.50]	0.0001	49
< 2.4	7	2.18 [1.74, 2.72]	< 0.00001	57
BMI				
≥ 25	4	2.77 [1.64–4.66]	0.0001	57
< 25	7	2.04 [1.69–2.47]	< 0.00001	48
FPG				
≥ 9	3	3.58 [1.81, 7.05]	0.0002	31
< 9	5	2.09 [1.68, 2.60]	< 0.00001	68
Diagnostic markers				
UACR and equivalents	6	2.37 [1.82, 3.08]	< 0.00001	41
eGFR	8	2.04 [1.67, 2.49]	< 0.00001	47

NLR, neutrophil-to-lymphocyte ratio; DN, diabetic nephropathy; OR, odds ratio; CI, confidence interval; BMI, body mass index; FPG, fasting plasma glucose; eGFR, estimated glomerular filtration rate; UACR, urinary albumin-to-creatinine ratio.

### Quality assessment

The quality of the studies included in the meta-analysis was evaluated using the Newcastle-Ottawa Quality Assessment Scale (NOS). This scale evaluates studies across 3 key domains: selection, comparability, and outcomes, with a total possible score of 9 points^41^. Studies achieving a score between 7 and 9 were deemed high-quality^42^. Two authors (NHY and TC) independently conducted the quality assessment, and any discrepancies were resolved through discussion with a third author (LH).

### Statistical analysis

Meta-analysis was conducted using Review Manager (version 5.4.1). For dichotomous data, Odds ratios (ORs) were calculated, while standardized mean differences (SMDs) were applied for continuous data. Results were reported with 95% confidence intervals (CIs). Heterogeneity for each outcome was assessed using the chi-squared (χ²) test (Cochran’s Q) and the I² index^43^, with a χ² *P* value < 0.1 or an I² value > 50% indicating substantial heterogeneity. A random-effects model based on the DerSimonian-Laird (DL) method was employed to estimate between-study variance and derive pooled ORs or SMDs. Sensitivity analyses were conducted for outcomes with at least 3 studies to assess the impact of individual studies on the overall effect size. Subgroup analyses, stratified by study design, geographic region, age, BMI, FPG, NLR cut-off values, and diagnostic markers, were undertaken to evaluate robustness and explore potential heterogeneity sources. Funnel plots were generated in Review Manager, and Egger’s regression test was performed using STATA version 15.1 (StataCorp, College Station, TX, USA) to assess publication bias for all outcomes^44^. In STATA, the “metabias” command was used to perform Egger’s test for small-study effects, with significance set at *P* < 0.05. Additionally, the trim-and-fill method was applied using STATA to impute missing studies for the affected outcomes, and Galbraith plots were generated to explore the impact of study heterogeneity. Potential dependence among subgroups from the same study was also considered. Several within-study contrasts sharing a common comparator were identified as outliers by Galbraith plots, and the results after excluding these heterogeneity-driving subgroups are presented in Supplementary Table S3. Removing these outliers markedly reduced heterogeneity while maintaining the overall direction of associations. All meta-analyses required a minimum of 3 independent studies for inclusion to ensure adequate statistical power. A *P* value < 0.05 was considered evidence of statistically significant publication bias. To avoid inappropriate pooling, DN occurrence, kidney function deterioration, and mortality were analyzed as independent outcomes, with SMDs and ORs calculated within each diagnostic definition (UACR, eGFR, or others) separately.

## Results

### Study characteristics

An initial search of the databases retrieved 762 articles, with 252 excluded due to duplicate records. After screening titles and abstracts, 433 additional studies were removed. Full-text evaluations were conducted on 77 studies, of which 38 were excluded, primarily for lacking relevant data required for incidence rate analysis. Ultimately, 39 studies were included in the meta-analysis, encompassing a total of 14,300 patients (Fig. 1).

Among the 39 included studies, 2 were conducted in North America^45,46^, 2 in Africa^47,48^, and the remaining 35 studies in Asia^2,3,15,34,35,49–78]^. Given that the vast majority of studies (35/39) originated from Asian populations, particularly East and South Asia, the pooled results should primarily be interpreted within this ethnic and geographic context. This regional concentration may limit the generalizability of the findings to non-Asian populations. The dataset included 34 case-control studies and 5 cohort studies. Due to stratified reporting by disease severity, 39 studies yielded 57 analyzable subgroups: 19 subgroups with continuous NLR comparing DN and DM, and 13 subgroups with continuous NLR comparing DN patients with kidney function deterioration and those with stable kidney function. Additionally, 14 subgroups provided ORs for DN occurrence, 4 provided ORs for incidence of kidney function deterioration, 5 provided ORs for mortality. Supplementary Table S1 presents the characteristics of the included studies. As clarified in the Methods, potential dependence among subgroups from the same study was examined using Galbraith plots, and heterogeneity-driving contrasts were excluded in sensitivity analyses (see Supplementary Table S3), which confirmed that the overall pooled estimates remained robust after adjustment.

### Risk of bias

All 39 studies included in our analysis scored between 6 and 9 for the NOS. Specifically, 11 studies achieved a score of 9, 21 studies scored 8, 6 studies scored 7, and 1 study scored 6. The majority of point deductions were due to inadequate adjustment for “comparability on the most important factors”, and primarily involving variables such as age and gender. Overall, the NOS results indicate a generally moderate-to-low risk of bias; however, potential residual confounding cannot be ruled out because several studies did not fully adjust for key clinical covariates (Supplementary Table S2).

### Meta-analysis results

#### Association between NLR as a continuous variable and DN risk

**Fig. 2:**
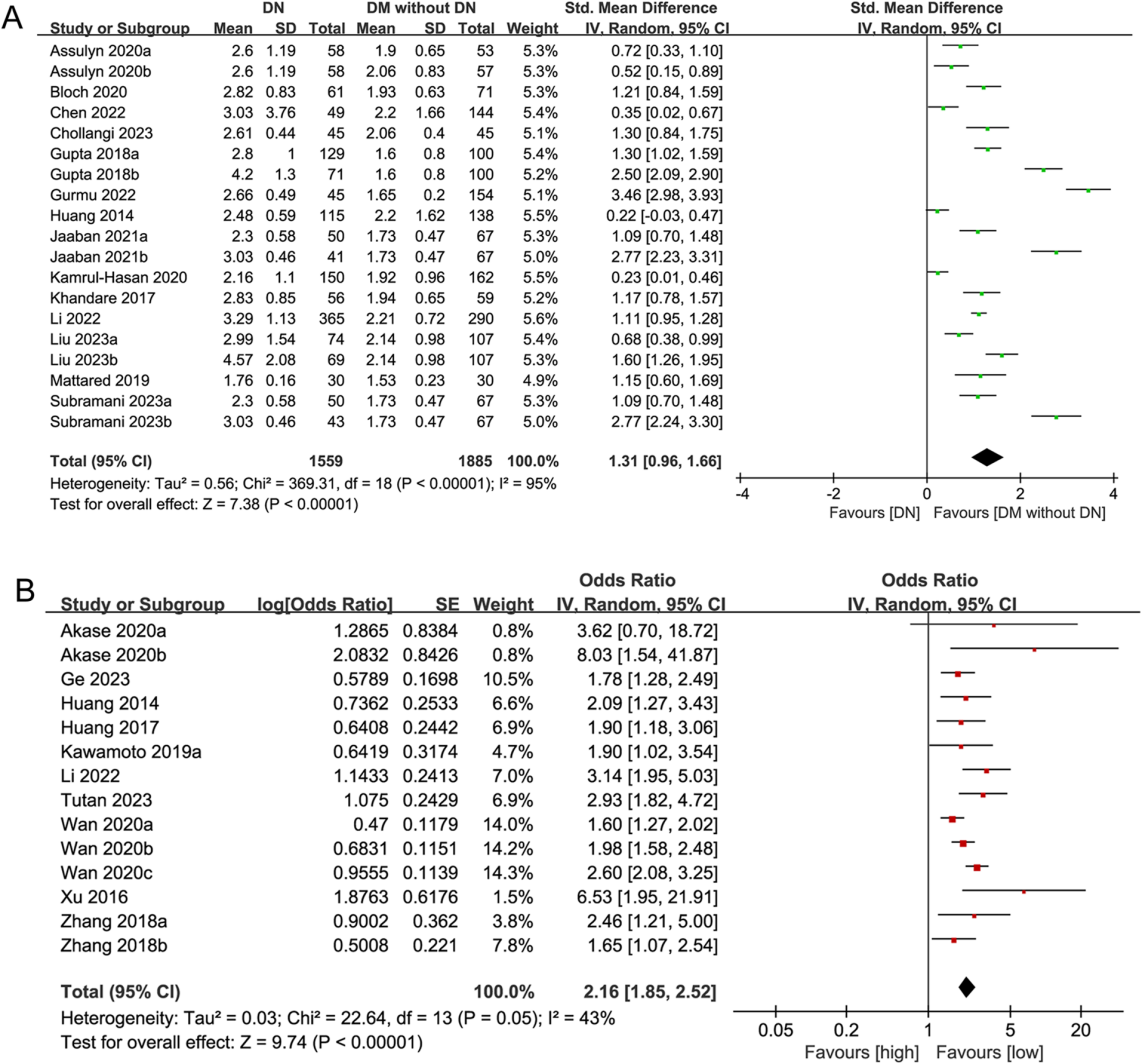
Forest plots for the association between NLR and DN risk. (**A**) Forest plot of NLR (continuous) vs. DN risk; (**B**) forest plot of NLR (categorical) vs. DN risk.

The association between NLR (continuous) and DN risk was analyzed across 14 case-control studies (19 subgroups) involving 3,444 participants (1559 DN cases vs. 1885 controls). A random-effects meta-analysis revealed significantly higher NLR levels in DN cases compared to controls, with a large effect size (SMD = 1.31, 95%CI 0.96–1.66, *P* < 0.00001) (Table 1; Fig. 2A). However, substantial heterogeneity (I² > 90%) was observed across studies, indicating that the pooled SMD should be interpreted as reflecting an overall trend rather than a precise estimate of effect size. Subgroup analyses stratified by region, age, NLR cutoff, BMI, FPG, and diagnostic markers (UACR vs. eGFR) showed significantly higher NLR levels in DN patients (all subgroup *P* values < 0.05), despite variations in SMD magnitudes. By region, the highest SMD was observed in South Asia, followed by West Asia and East Asia (1.43 vs. 1.25 vs. 0.79). By age, patients aged ≥ 60 years had a lower SMD than those aged < 60 years (0.92 vs. 1.42). By NLR cutoff, patients with a cutoff ≥ 2.4 had a lower SMD than those with a cutoff < 2.4 (0.81 vs. 1.54). By BMI, patients with BMI ≥ 25 kg/m² had a lower SMD than those with BMI < 25 kg/m² (1.24 vs. 1.61). By FPG, patients with FPG < 9 mmol/L had a lower SMD than those with FPG ≥ 9 mmol/L (1.12 vs. 1.44). By diagnostic markers, the SMD value for eGFR was lower than that for UACR and equivalents (0.71 vs. 1.47).

#### Association between NLR as a categorical variable and DN risk

The association between NLR (categorical) and DN risk was evaluated in 3 cohort studies (7 subgroups) and 7 case-control studies (7 subgroups) involving 7,967 participants. Consistent with the findings from the analysis of NLR as a continuous variable, high NLR values (vs. low NLR values) were significantly associated with an increased risk of DN in the overall analysis (OR = 2.16, 95%CI: 1.85–2.52, *P* < 0.00001) (Table 3; Fig. 2B). Subgroup analyses stratified by study design, region, age, NLR cutoff, BMI, FPG, and diagnostic markers (UACR vs. eGFR) consistently revealed a significantly higher risk of DN in DM patients with high NLR values compared to those with low NLR values, although variations in OR magnitudes. By study design, the OR was higher in case-control studies than in cohort studies (2.36 vs. 1.85). By region, Japanese patients exhibited a higher OR than Chinese patients (2.84 vs. 2.08). By age, patients aged ≥ 60 years had a higher OR than those aged < 60 years (2.29 vs. 1.94). By NLR cutoff, patients with a cutoff ≥ 2.4 showed a higher OR than those with a cutoff < 2.4 (2.30 vs. 2.18). By BMI, patients with BMI ≥ 25 kg/m² had a higher OR than those with BMI < 25 kg/m² (2.77 vs. 2.04). By FPG, patients with FPG ≥ 9 mmol/L had a higher OR than those with FPG < 9 mmol/L (3.58 vs. 2.09). By diagnostic markers, the OR value for eGFR was slightly lower than that for UACR and equivalents (2.04 vs. 2.37).

**Table 3:** Meta-analyses between NLR as a continuous variable and kidney function deterioration.

Groups/subgroups	*N*	SMD [95%CI]	*P* value	I^2^
Total	13	1.02 [0.77, 1.26]	< 0.00001	80%
Mean/median age				
≥ 60	6	1.12 [0.71, 1.53]	< 0.00001	85%
< 60	7	0.94 [0.62, 1.26]	< 0.00001	75%
BMI				
≥ 25	5	0.80 [0.38, 1.21]	0.0002	85%
< 25	5	1.16 [0.77, 1.55]	< 0.00001	79%
Diagnostic markers				
eGFR	4	0.83 [0.65, 1.01]	< 0.00001	0%
UACR	6	0.82 [0.46, 1.19]	< 0.0001	82%
Others	3	1.28 [1.03, 1.53]	< 0.00001	0%

NLR, neutrophil-to-lymphocyte ratio; SMD, standardized mean difference; CI, confience interval; BMI, body mass index; eGFR, estimated glomerular filtration rate; UACR, urinary albumin-to-creatinine ratio.

#### Association between NLR as a continuous variable and DN deterioration

**Fig. 3:**
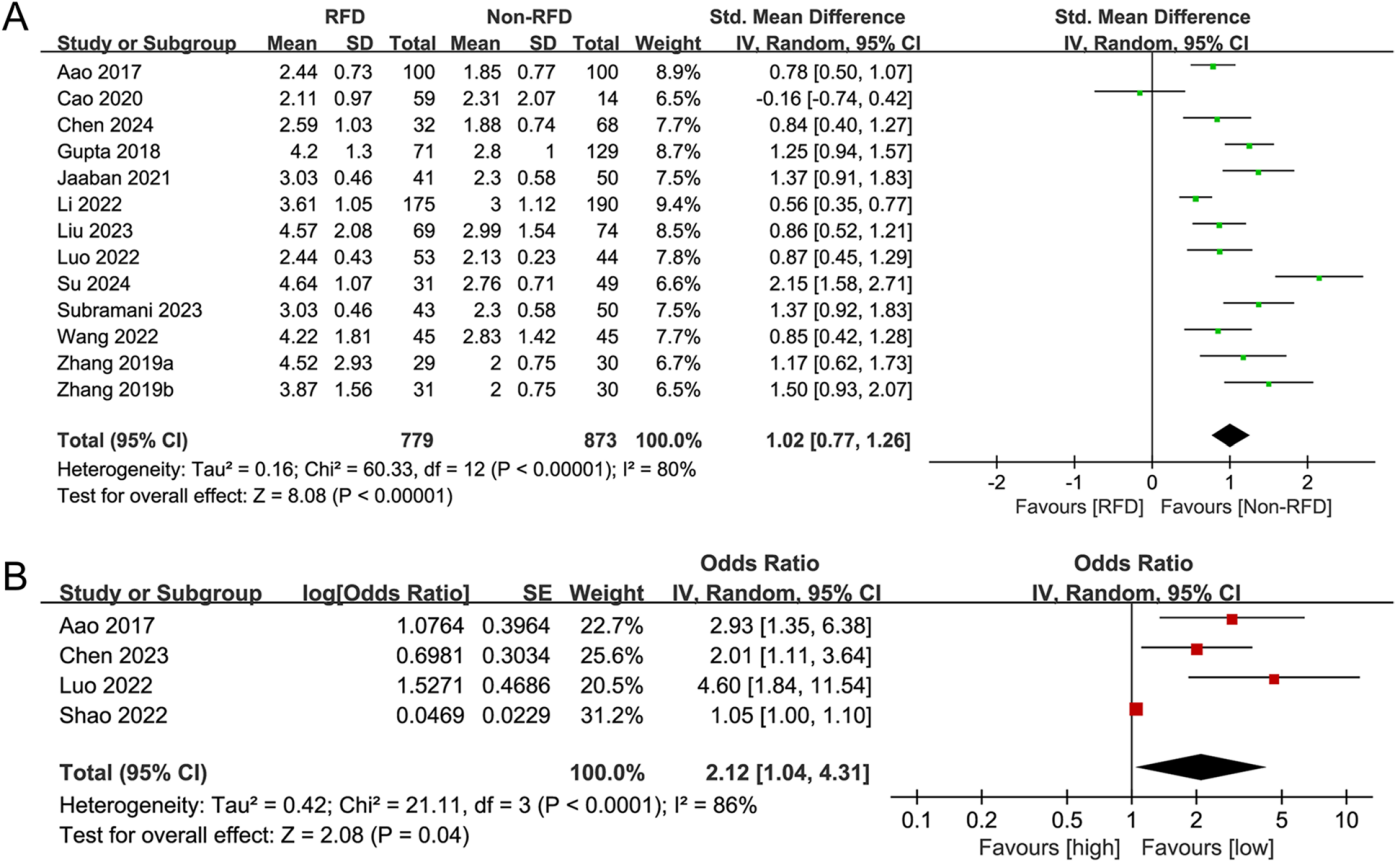
Forest plots for the association between NLR and kidney function deterioration. (**A**) Forest plot of NLR (continuous) vs. kidney function deterioration; (**B**) Forest plot of NLR (categorical) vs. kidney function deterioration.

The association between NLR (continuous) and kidney function deterioration in DN was analyzed across 12 case-control studies (13 subgroups) involving 779 DN patients with kidney function deterioration and 873 stable DN controls. The overall analysis revealed that NLR levels were significantly higher in DN patients with kidney function deterioration compared to stable DN controls (SMD = 1.02, 95%CI 0.77–1.26, *P* < 0.00001) (Table 3; Fig. 3A). Subgroup analyses stratified by age, BMI, and kidney function assessment parameters consistently demonstrated higher NLR levels in DN patients with kidney function deterioration, although the SMD values varied across subgroups. By age, patients aged < 60 years exhibited a lower SMD than those aged ≥ 60 years (0.94 vs. 1.12). By BMI, patients with BMI ≥ 25 kg/m² had a lower SMD than those with BMI < 25 kg/m² (0.80 vs. 1.16). By kidney function assessment parameters, the SMD values for eGFR and UACR were lower than those for other kidney function assessment parameters (0.83 vs. 0.82 vs. 1.28).

#### Association between NLR as a categorical variable and DN deterioration

The association between NLR (categorical) and kidney function deterioration in DN was evaluated across 4 case-control studies (4 subgroups) involving 290 DN patients with kidney function deterioration and 254 stable DN controls. Consistent with the findings for NLR as a continuous variable, high NLR values (vs. low NLR values) were significantly associated with an increased risk of kidney function deterioration in DN (OR = 2.12, 95%CI 1.04–4.31, *P* = 0.04) (Fig. 3B).

#### Association between NLR as a categorical variable and DN mortality

**Fig. 4:**
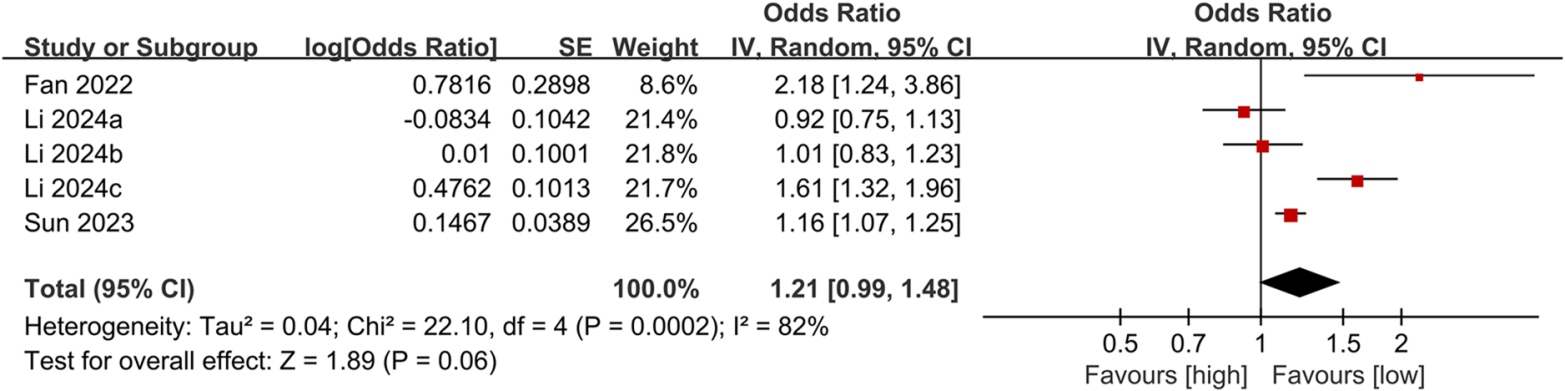
Forest plot for the association between NLR (categorical) and mortality.

The association between NLR (categorical) and DN mortality was assessed in 3 independent studies (5 subgroups) involving 2,220 participants (1239 survivors vs. 971 non-survivors). The analysis showed that high NLR values (vs. low NLR values) were marginally insignificantly associated with an increased mortality risk (OR = 1.21, 95%CI 0.99–1.48, *P* = 0.06) (Fig. 4).

### Sensitivity analysis

**Fig. 5:**
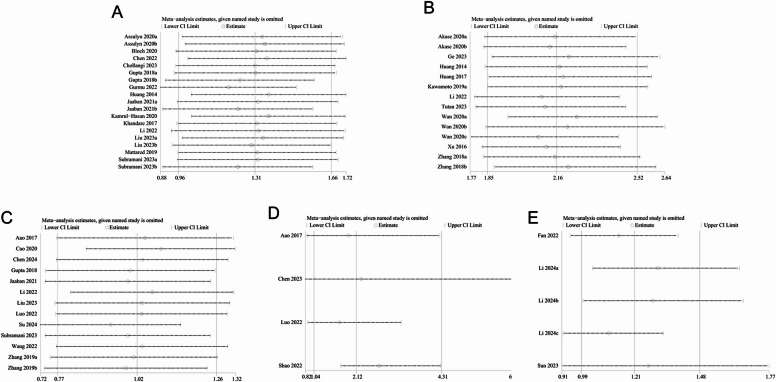
Sensitivity analyses. (**A**) Sensitivity analysis of NLR (continuous) and DN; (**B**) Sensitivity analysis of NLR (categorical) and DN; (**C**) Sensitivity analysis of NLR (continuous) and kidney function deterioration; (**D**) Sensitivity analysis of NLR (categorical) and kidney function deterioration; (**E**) Sensitivity analysis of NLR and mortality.

Sensitivity analysis was conducted to assess the robustness of the findings on the association between NLR and DN, as well as its clinical outcomes. The results demonstrated that the effect sizes remained stable within the initial ranges for the associations between NLR (continuous) and DN risk (Fig. 5A), NLR (categorical) and DN risk (Fig. 5B), as well as NLR (continuous) and kidney function deterioration (Fig. 5C) after sequentially excluding individual studies. However, significant changes in effect sizes were observed for both the association of NLR (categorical) and kidney function deterioration (Fig. 5D), as well as NLR (categorical) and DN mortality (Fig. 5E). These findings suggest that the results for NLR and mortality should be interpreted with caution, as the sensitivity analysis showed variability with the inclusion or exclusion of individual studies.

### Publication bias

**Fig. 6:**
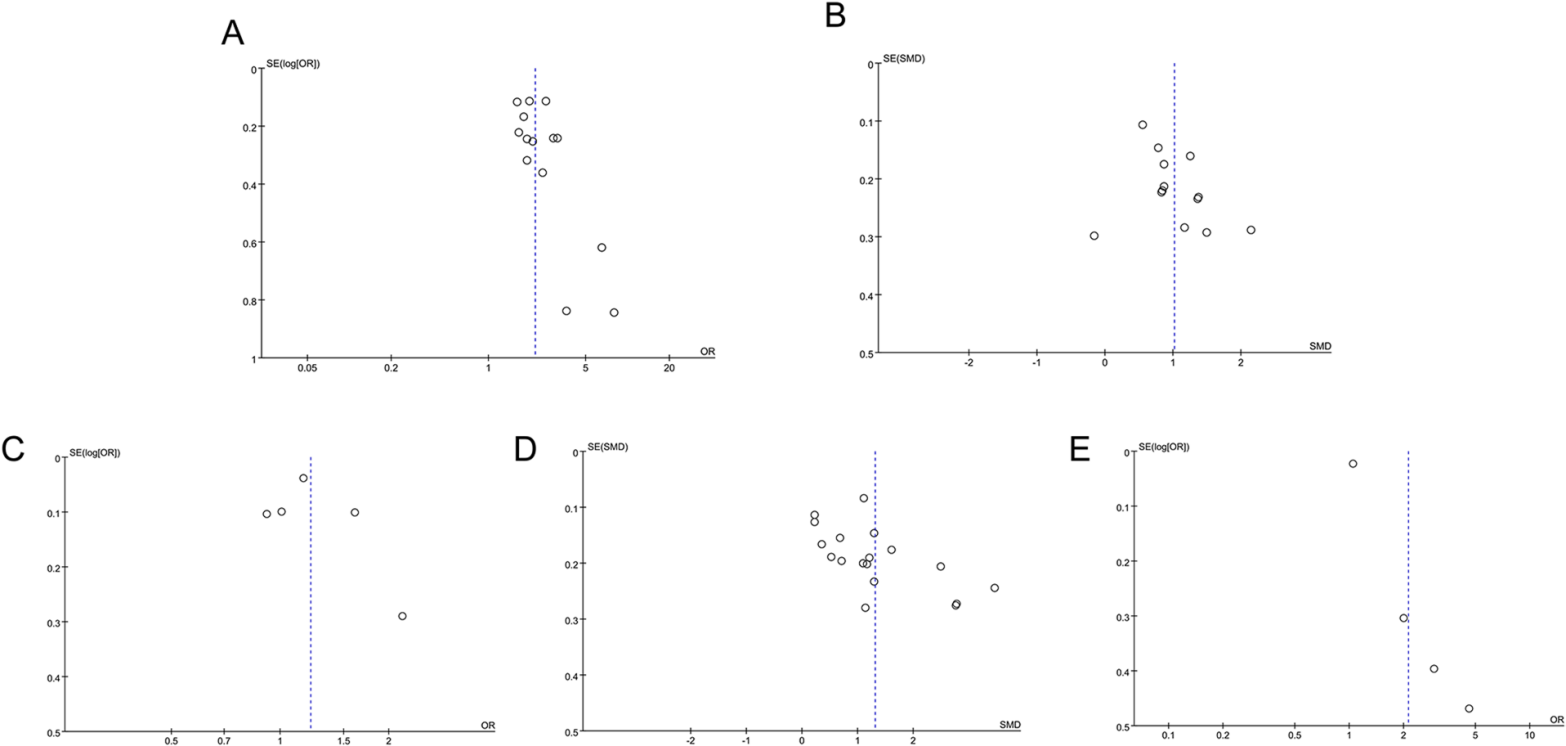
Funnel plots for the evaluation of publication bias. (**A**) Funnel plot for the evaluation of publication bias for NLR (categorical) and DN; (**B**) Funnel plot for the evaluation of publication bias for NLR (continuous) and kidney function deterioration; (**C**) Funnel plot for the evaluation of publication bias for NLR and mortality; (**D**) Funnel plot for the evaluation of publication bias for NLR (continuous) and DN; (**E**) Funnel plot for the evaluation of publication bias for NLR (categorical) and kidney function deterioration.

Publication bias was assessed using funnel plots and Egger’s test. No significant publication bias was observed in the analyses of NLR (categorical) and DN risk (*P* = 0.095) (Fig. 6A), NLR (continuous) and kidney function deterioration (*P* = 0.099) (Fig. 6B), and NLR (categorical) and DN mortality (*P* = 0.648) (Fig. 6C). However, significant publication bias was detected in the analysis of NLR (continuous) and DN risk (*P* = 0.023) (Fig. 6D) as well as NLR (categorical) and kidney function deterioration (*P* = 0.011) (Fig. 6E). To correct for this bias, the trim-and-fill method was employed. No missing studies were identified for the association between NLR (continuous) and DN risk, and the results remained consistent. In the analysis of NLR (categorical) and kidney function deterioration, the trim-and-fill method identified 6 missing studies. Their inclusion did not significantly alter the overall results (OR = 1.055, 95%CI: 1.009–1.103, *P* = 0.019), but it is important to note that the addition of these studies reinforces the caution in interpreting these findings due to the potential for publication bias in the original data.

### Heterogeneity analysis

Significant heterogeneity was observed in both overall analyses: NLR (continuous) associated with DN risk (Table 1) and NLR associated with kidney function deterioration (Table 3). Nine studies (Assulyn 2020 B, Chen 2022, Gupta 2018 B, Gurmu 2022, Huang 2014, Jaaban 2021 B, Kamrul-Hasan 2020, Liu 2023 B, Subramani 2023 B) were identified as the primary sources of heterogeneity in the analysis of NLR (continuous) and DN risk, while 3 studies (Su 2024, Li 2022, Cao 2020) were the main contributors to heterogeneity in the analysis of NLR (continuous) and kidney function deterioration. After excluding these outlier studies, heterogeneity was significantly reduced or eliminated, with no substantial changes in the pooled results: for NLR (continuous) and DN, SMD = 1.08 (95%CI: 0.94–1.21, *P* < 0.00001, *I*^2^ = 39%); for NLR (continuous) and kidney function deterioration, SMD = 1.05 (95%CI: 0.88–1.22, *P* < 0.00001, *I*^2^ = 39%). Significant heterogeneity was also noted in some subgroup analyses, but its removal did not significantly alter the results (Supplementary Table S3).

## Discussion

This meta-analysis comprehensively evaluated the association between the NLR and DN across the disease spectrum, including occurrence, progression, and mortality, based on 39 studies involving 14,300 individuals. The findings suggest that elevated NLR levels, both as continuous and categorical variables, are significantly associated with increased DN risk and kidney function deterioration. Although the association with DN-related mortality did not reach statistical significance, a marginal trend was observed, indicating the need for further investigation. Compared with previous meta-analyses^36,37^ that mainly addressed the association between NLR and the early occurrence of DN, our study additionally examined kidney function deterioration and mortality, providing a broader perspective on the potential predictive value of NLR across the full clinical course of DN. Furthermore, by incorporating both continuous and categorical NLR data and conducting subgroup analyses for age, BMI, and glycemic control, this study offers complementary evidence that may enhance risk stratification strategies beyond those reported in earlier reviews.

In subgroup analyses, the associations between NLR and DN varied across several clinical characteristics, including age, BMI, and FPG; however, these results should be interpreted cautiously due to the limited number of studies and high heterogeneity within subgroups. For BMI, participants with BMI ≥ 25 kg/m² showed smaller SMDs but larger ORs for DN risk compared with those with lower BMI, and a similar trend was observed for age (≥ 60 vs. < 60 years). These discrepancies likely arise from methodological rather than biological factors—such as differences in data scaling, cutoff definitions, and study composition—whereby continuous analyses may underestimate effects due to broader variance, while categorical models amplify relative odds around risk thresholds. For FPG, both the SMD and OR were higher in the FPG ≥ 9 mmol/L subgroup than in < 9 mmol/L (SMD = 1.44 vs. 1.12; OR = 3.58 vs. 2.09), indicating that poorer glycemic control may strengthen the association between NLR and DN risk. Nonetheless, this observation remains exploratory given the small number of studies and substantial heterogeneity (I² > 85%). By diagnostic markers, the SMD and OR based on eGFR were lower than those based on UACR and equivalents (0.71 vs. 1.47; 2.04 vs. 2.37), reflecting measurement differences and population composition rather than superiority of one indicator. Collectively, these subgroup findings are exploratory and emphasize the need for larger, standardized studies to confirm potential modifying effects of age, BMI, and glycemic control on the relationship between NLR and DN.

The significant association between elevated NLR levels and the risk and progression of DN may reflect a series of interconnected mechanisms directly supporting our meta-analytic findings, linking systemic inflammation, immune dysregulation, oxidative stress, and fibrotic remodeling. Our meta-analysis demonstrated consistent elevations of NLR across different DN stages, supporting its role as both a biomarker and a mediator in diabetic kidney injury. First, hyperglycemia-induced oxidative stress leads to excessive reactive oxygen species production and activation of the NF-κB signaling cascade, promoting secretion of IL-6, TNF-α, and MCP-1, which further recruit neutrophils and perpetuate kidney inflammation^79–86^. Second, diabetes impairs lymphocyte-mediated immune regulation and favors myeloid lineage dominance due to uremic toxins such as indoxyl sulfate, amplifying chronic inflammation and sustaining high NLR levels^87,88^. Third, new evidence highlights the contribution of neutrophil extracellular traps (NETs) to DN pathogenesis. High glucose can induce excessive NET formation, and the released extracellular DNA–histone complexes trigger pyroptosis in glomerular endothelial cells and activate the NLRP3 inflammasome, aggravating glomerular and tubular injury^89^. Fourth, crosstalk between the NF-κB and PI3K/AKT pathways amplifies inflammatory signaling, while the NLRP3 inflammasome mediates IL-1β and IL-18 release, promoting tubular cell apoptosis and matrix accumulation^90,91^. Finally, transforming growth factor-β (TGF-β)–driven SMAD signaling accelerates kidney fibrosis and extracellular matrix deposition, leading to irreversible nephron loss^92,93^. In accordance with recent findings^94^, these inflammatory and oxidative pathways also intersect with insulin resistance and mitochondrial dysfunction, further linking metabolic stress to immune activation. Collectively, these findings indicate that the elevated NLR observed in our meta-analysis reflects not merely a statistical correlation but a mechanistic marker of sustained inflammation and tissue remodeling driving DN onset and progression.

Heterogeneity assessment, publication bias detection, and sensitivity analyses were conducted to examine result reliability. Heterogeneity analysis revealed significant variability in NLR (continuous) and DN risk as well as kidney function deterioration. Despite extensive subgroup and sensitivity analyses, high I² values persisted, suggesting that the pooled SMD and OR estimates represent directional rather than precise quantitative associations and should therefore be interpreted with caution. This variability arises from multiple factors including inconsistencies in DN diagnostic criteria, differences in DN severity stages across studies, methodological disparities (particularly in confounder adjustment as identified by NOS evaluation), population diversity, variability in glycemic control strategies, differential prevalence of complications, and heterogeneity in treatment regimens. Importantly, to avoid inappropriate pooling, DN occurrence, kidney function deterioration, and mortality were analyzed as independent outcomes, and subgroup analyses were further stratified by diagnostic markers (UACR and equivalents, eGFR, and others) to minimize methodological heterogeneity. Notably, exclusion of outlier studies reduced heterogeneity without significantly altering pooled estimates, supporting result stability. While NOS assessment confirmed high methodological quality in most studies (38/39 scoring ≥ 7/9), inconsistent adjustment for key confounders (e.g., hypertension, duration of diabetes and HbA1c) was observed in the comparability domain (only 21/39 studies adjusted for most primary and secondary confounders). This may introduce residual confounding, though heterogeneity adjustment and bias correction suggest it did not substantially alter our conclusions. Publication bias evaluation showed absence of bias in most analyses, though it was detected in NLR (continuous) and DN risk, as well as NLR (categorical) and kidney function deterioration. The trim-and-fill adjustment for these outcomes did not significantly change effect estimates, further confirming result validity. Sensitivity analyses demonstrated that the association between NLR (categorical) and kidney function deterioration was particularly influenced by variations in outcome definitions, especially the stringent criteria introduced by Shao et al. ^56^ For mortality outcomes, the relationship proved sensitive to NLR cutoff thresholds, with higher values (> 3) yielding more consistent risk predictions. Taken together, the stability of results after accounting for methodological limitations (including those identified by NOS), heterogeneity adjustment and bias correction reinforces the validity of our conclusions, while the observed sensitivity to outcome definitions and NLR thresholds highlights the necessity of standardized methodologies in future studies.

Our findings on the association between NLR and DN progression raise important considerations for clinical practice. While traditional biomarkers such as UACR and eGFR remain cornerstone tools for DN diagnosis and monitoring, NLR offers additional value as an easily obtainable inflammatory marker that may enhance risk stratification. At present, NLR behaves more as a continuous inflammatory indicator rather than a marker with a universally defined clinical cutoff, as thresholds varied across studies based on individual ROC analyses. Rather than defining absolute thresholds, NLR should currently be regarded as a supportive indicator to help recognize patients at potentially higher risk who might warrant closer follow-up or earlier intervention. The wide variability in cutoff values reported across studies underscores the need for standardized protocols to establish clinically validated thresholds. Moreover, the non-specific nature of NLR necessitates its integration with existing biomarkers for a more comprehensive evaluation of DN progression.

Several limitations should be acknowledged. First, most included studies (35/39) were conducted in Asian populations, which may restrict the generalizability of our findings to other ethnic groups. The applicability of these results to non-Asian populations, such as Caucasians and Hispanics, should therefore be interpreted with caution. Additionally, it should be noted that all included studies used clinically diagnosed DKD rather than biopsy-confirmed DN; the term “DN” in this study is used to maintain consistency with the terminology adopted in the original literature. Second, significant heterogeneity was observed in some analyses, likely due to variations in diagnostic criteria, DN severity, and inconsistent adjustment for key confounders (e.g., HbA1c, blood pressure, diabetes duration). In particular, the inclusion of multiple subgroups from the same study as independent contrasts may have introduced methodological bias. This issue was addressed through outlier detection and exclusion using Galbraith plots (Supplementary Table S3), which reduced heterogeneity without altering the main conclusions. Third, the results for mortality and kidney function deterioration (categorical) were not robust in sensitivity analyses, suggesting these specific associations should be interpreted with caution. The limited number of studies and relatively short follow-up periods likely contributed to this instability. Finally, several studies lacked adequate adjustment for major confounders, leaving the possibility of residual bias. Future research with more diverse cohorts, standardized diagnostic criteria, longer follow-up, and rigorous confounder control is needed to validate and refine these findings. In summary, these limitations introduce considerable uncertainty, emphasizing the need for larger, more diverse cohorts, longer follow-up periods, and more standardized methodologies to confirm these findings and validate the predictive role of NLR in DN.

In conclusion, this meta-analysis provides an updated synthesis of current evidence on the association between NLR and the occurrence, progression, and mortality of diabetic nephropathy. While elevated NLR appears to be consistently associated with higher risks of DN onset and kidney function decline, these results should be interpreted with caution given the high heterogeneity, methodological variability, and predominance of Asian populations among included studies. The findings highlight the potential of NLR as a promising inflammatory biomarker for DN risk stratification, but further large-scale, multiethnic, and methodologically standardized studies are needed to validate these preliminary observations and establish clinically applicable thresholds.

## Supplementary Information

Below is the link to the electronic supplementary material.

Supplementary Material 1

## Data Availability

The data used to support the findings of this study are included within the article.
