# Supplementary material for: The predictive value of neutrophil-to-lymphocyte ratio for the occurrence, progression, and mortality of diabetic nephropathy: a systematic review and meta-analysis
Source: Sci Rep. 2026 Jan 5;16:1099. doi: 10.1038/s41598-025-30680-4 (PMC12789594; doi:10.1038/s41598-025-30680-4)
Supplement: Supplementary file 1 — Supplementary Material 1 [file 41598_2025_30680_MOESM1_ESM.pdf]

**Supplementary Table S1.** The characteristics of the included studies.

| Author | Study period | Region | Diagnostic markers | Study design | No. of patients | Gender | | Mean/median age | BMI | FPG | eGFR | NLR cutoff |
|---|---|---|---|---|---|---|---|---|---|---|---|---|
| | | | | | | M | F | | | | | |
| Chen 2022 | 2019.8-2019.12 | China | UACR/eGFR | Case-control | 183 | 144 | 39 | 54.75±11.54 | NA | NA | NA | 1.56 |
| Olores 2023 | 2018.1-2018.7 | Filipino | Hemodialysis | Cohort (All) | 63 | 35 | 28 | 63.38±11.107 | NA | NA | NA | |
| | | | | Cohort I (NLR<3.5) | 39 | 21 | 18 | 63.51±10.933 | 24.87±5.29 | NA | NA | 3.5 |
| | | | | Cohort II (NLR≥3.5) | 24 | 14 | 10 | 63.17±11.620 | 25.36±4.50 | NA | NA | |
| Li 2022 | 2018.1-2021.1 | China | UACR | Case-control I (DM) | 290 | 154 | 136 | 59.04±10.63 | 24.91±3.29 | 8.54±2.32 | 102.26±12.40 | 2.46 |
| | | | | Case-control II (DN1) | 190 | 103 | 87 | 60.23±11.39 | 25.09±3.57 | 8.76±2.06 | 96.75±8.84 | |
| | | | | Case-control III (DN2) | 175 | 94 | 81 | 61.13±9.66 | 25.25±3.05 | 9.12±2.04 | 76.65±6.68 | |
| Kawamoto 2019 | 2017.4-2017.6 | Japan | eGFR | Case-control I (DM) | 265 | 140 | 125 | 72±11 | 24.1±3.7 | NA | 71.8±7.5 | NA |
| | | | | Case-control II (DN1) | 77 | 37 | 40 | 81±7 | 25.0±3.5 | NA | 52.8±4.3 | |
| | | | | Case-control III (DN2) | 44 | 22 | 22 | 81±9 | 24.9±4.0 | NA | 37.7±4.7 | |
| Suvarna 2023 | 2021.1-2021.6 | India | NA | Case-control I (DM) | 100 | 65 | 35 | 55.2±12 | NA | NA | NA | NA |
| | | | | Case-control II (DN) | 100 | 64 | 36 | 59.8±10 | NA | NA | NA | |
| Wan 2020 | 2018 | China | UACR/eGFR | Cohort I (NLR≤1.382) | 1194 | 464 | 730 | 66.16±8.38 | 24.67±3.51 | 7.46±2.13 | 94.17±14.70 | 1.382 |
| | | | | Cohort II | 1211 | 530 | 681 | 67.06±8.61 | 25.07±3.60 | 7.66±2.40 | 92.82±15.57 | 1.777 |

| | | | | | | | | | | | | |
|---|---|---|---|---|---|---|---|---|---|---|---|---|
| | | | | (1.382<NLR≤1.777) | | | | | | | | |
| | | | | Cohort III (1.777<NLR ≤2.319) | 1198 | 577 | 621 | 66.69±8.40 | 25.26±3.58 | 7.90±2.45 | 92.35±16.51 | 2.319 |
| | | | | Cohort IV (NLR>2.319) | 1194 | 640 | 554 | 68.73±9.04 | 24.84±3.67 | 8.06±2.77 | 87.30±20.12 | |
| Liu 2023 | 2020.1-2021.8 | China | UACR/eGFR | Case-control I (DM) | 107 | 53 | 54 | 57. 1±11.3 | 23. 55±3.20 | 10. 08±3.20 | NA | |
| | | | | Case-control II (DN1) | 74 | 41 | 33 | 60. 6±12.7 | 24. 00±3.96 | 6. 11±2.76 | NA | NA |
| | | | | Case-control III (DN2) | 69 | 36 | 33 | 58. 5±10.5 | 23. 78±3.31 | 7. 46±3.20 | NA | |
| Tutan 2023 | 2021.1-2023.3 | Turkey | UPCR | All | 327 | 148 | 179 | 61 (23-92)# | NA | 14.22 (3.39-37.44) # | NA | |
| | | | | Case-control I (DM) | 219 | 101 | 118 | 60 (23-85)# | NA | 14 (4.72-30.28) # | NA | 1.93 |
| | | | | Case-control II (DN) | 108 | 47 | 61 | 65 (25-92)# | NA | 14.89 (3.39-37.44) # | NA | |
| Zhang 2018 | 2009-2017 | China | eGFR | All | 247 | 171 | 76 | 51±8.89 | NA | NA | 56.53±34.01 | |
| | | | | Cohort I (NLR<2.42) | 122 | 80 | 42 | 52±9.63 | NA | NA | 66.78±37.41 | 2.42 |
| | | | | Cohort II (NLR≥2.42 ) | 125 | 91 | 34 | 51±8.15 | NA | NA | 50.03±27.58 | |
| Kamrul-Hasan 2020 | 2018.7-2019.6 | Bangladesh | UACR/eGFR | Case-control I (DM) | 162 | 81 | 81 | 48.6±9.6 | 26.6 ±4.1 | 9.2 ±3.0 | 89.5±17.0 | NA |
| | | | | Case-control II (DN) | 150 | 60 | 90 | 51.9±11.9 | 26.5 ±4.8 | 10.3 ±4.1 | 75.6±25.1 | |

| | | | | | | | | | | | | |
|---|---|---|---|---|---|---|---|---|---|---|---|---|
| Chollangi 2023 | 2020.12-2022.7 | India | UAC | Case-contro I (DM) | 45 | 19 | 26 | 62.40±9.9 | NA | NA | NA | 2.13 |
| | | | | Case-contro II (DN) | 45 | 18 | 27 | 62.09±9.6 | NA | NA | NA | |
| Mattared 2019 | 2017.3-2017.9 | Egypt | UAC | Case-control I (DM) | 30 | NA | NA | 60.40±9.98 | NA | 11.19±3.10 | 99.13±12.72 | NA |
| | | | | Case-control II (DN) | 30 | NA | NA | 59.10±8.36 | NA | 12.15±3.10 | 93.37±9.33 | |
| Ciray 2015 | 2013.10-2014.2 | Turkey | UACR/eGFR | All | 114 | 41 | 73 | 59.7±11.3 | 30.3±4.91 | NA | NA | NA |
| | | | | Case-control I (DM) | 52 | 41 | 73 | 56.9±10.1 | 30.9±4.61 | NA | 89.8±21.3 | |
| | | | | Case-control II (DN) | 62 | | | 62.1±11.7 | 31.1±7.02 | NA | 67.2±28.2 | |
| Singh 2021 | 2019.6-2020.6 | India | UAC | Case-control I (DM) | 178 | 109 | 69 | 55.59±10.64 | NA | NA | 98.78±11.88 | 3.28 |
| | | | | Case-control II (DN) | 146 | 86 | 60 | 56.66±10.23 | NA | NA | 35.27±29.79 | |
| Jaaban 2021 | 2017-2019 | Syria | UACR | Case-control I (DM) | 67 | 39 | 28 | 54±10 | 27.66±2.4 | NA | 107.7±14.75 | 2.2 |
| | | | | Case-control II (DN1) | 50 | 29 | 21 | 58±7 | 27.27±2.37 | NA | 87.04±12.2 | |
| | | | | Case-control III (DN2) | 41 | 24 | 17 | 61±6 | 27.5±1.39 | NA | 70.32±7.23 | |
| Gurmu 2022 | 2019.10-2020.4 | Ethiopia | UAC | Case-control I (DM) | 154 | 71 | 83 | 53.85±11.900 | 24.87±1.47 | 10.81±1.56 | NA | NA |
| | | | | Case-control II (DN) | 45 | 17 | 28 | 60.36±10.30 | 25.35±1.77 | 11.50±4.19 | NA | |
| Huang 2014 | 2013.1-2014.1 | China | UAE | Case-control | 253 | 113 | 140 | 50.32±10.24 | 24.41±3.73 | NA | NA | NA |
| Assulyn 2020 | 2014-2017 | Israel | UAC | Case-control I (DM1) | 53 | 24 | 29 | 64±11 | 28.81±5.64 | NA | NA | 2.54 |
| | | | | Case-control II (DM2) | 57 | 27 | 30 | 61±10 | 29.06±4.73 | NA | NA | |
| | | | | Case-control III (DN) | 58 | 33 | 25 | 67±10 | 29.80±4.97 | NA | NA | |

| | | | | | | | | | | | | |
|---|---|---|---|---|---|---|---|---|---|---|---|---|
| Azab 2012 | 2007.12-2009.12 | America | eGFR | Cohort I (NLR<1.6) | 110 | 33 | 77 | 56.05±12.06 | 31.53±7.76 | NA | 88.44±26.01 | 1.6 |
| | | | | Cohort II (1.6≤NLR≤2.36) | 115 | 40 | 75 | 60.03±11.47 | 31.45±7.83 | NA | 76.64±27.56 | 2.3 |
| | | | | Cohort III (NLR>2.36) | 113 | 49 | 64 | 58.18±10.20 | 33.91±8.19 | NA | 77.71±22.64 | |
| Huang 2017 | 2014.1-2015.6 | China | UACR | Case-control I (DM) | 134 | 72 | 62 | 54.22±10.27 | 24.20±2.77 | NA | NA | |
| | | | | Case-control II (DN1) | 74 | 48 | 26 | 55.46±10.78 | 25.80±3.88 | NA | NA | 1.758 |
| | | | | Case-control III (DN2) | 113 | 66 | 47 | 60.03±10.31 | 24.89±3.47 | NA | NA | |
| Akase 2020 | 2017.4-2017.6 | Japan | eGFR | Cohort I (0.65≤ NLR≤1.57) | 120 | 62 | 58 | 73±11 | 25.2±3.8 | NA | 67.7±12.7 | 0.65 |
| | | | | Cohort II ( 1.58 ≤ NLR≤2.32) | 119 | 60 | 59 | 75±10 | 24.8±3.3 | NA | 63.4±13.9 | 1.58 |
| | | | | Cohort III (2.33≤ NLR≤15.3) | 119 | 62 | 57 | 76±10 | 23.3±3.8 | NA | 61.8±15.4 | 2.33 |
| Bloch 2020 | 2018.2-2020.2 | Pakistan | UAC | Case-control I (DN) | 61 | 30 | 31 | 53.00±11.23 | NA | 9.58±2.28 | 85.70±27.7 | NA |
| | | | | Case-control II (DM) | 71 | 38 | 33 | 51.05±11.21 | NA | 8.84±2.28 | 95.99±27.79 | |
| Subramani 2023 | 2021.11-2022.4 | India | UACR | Case-control I (DM) | 67 | 39 | 28 | 54±10 | 27.66±2.4 | NA | 107.7±14.75 | |
| | | | | Case-control II (DN1) | 50 | 29 | 21 | 58±7 | 27.27±2.37 | NA | 87.04±12.2 | 2.2 |
| | | | | Case-control III (DN2) | 41 | 24 | 17 | 61±6 | 27.5±1.39 | NA | 70.32±7.23 | |
| Khandare 2017 | 2015.3-2016.3 | India | UAE | Case-control I (DN) | 56 | 25 | 31 | 52.29±11.45 | 26.25±4.41 | 9.64±2.33 | 85.71±27.72 | NA |
| | | | | Case-control II (DM) | 59 | 26 | 33 | 50.05±11.29 | 26.07±4.11 | 8.9±2.33 | 96.2±28.23 | |

| | | | | | | | | | | | | |
|---|---|---|---|---|---|---|---|---|---|---|---|---|
| Gupta 2018 | 2018.4-2018.6 | India | UACR | Case-control I (DM) | 100 | | | 48.6±4.2 | 24.7±3.4 | NA | 118.2±32.4 | |
| | | | | Case-control II (DN1) | 129 | 162 | 138 | 47.6±5.8 | 24.1±4.1 | NA | 101±28.2 | NA |
| | | | | Case-control III (DN2) | 71 | | | 48.2±54.8 | 23.2±3.9 | NA | 88.0±34.2 | |
| Ge 2023 | 2019.10-2022.1 | China | UACR | Case-control I (DM) | 273 | 187 | 86 | 56 (49, 64)* | 24. 9±3.2 | 8.14 (6.51, 10.26)* | 106.52 (100.18, 116.27)* | |
| | | | | Case-control II (DN1) | 120 | 83 | 37 | 57 (49, 66)* | 26. 0±3.1 | 8.59 (6.37, 11.40)* | 104. 07 (92. 74, 115.84)* | NA |
| | | | | Case-control III (DN2) | 70 | 48 | 22 | 59 (49, 65)* | 25. 6±3.7 | 7.52 (5.57, 10.23)* | 73. 50 (27.83, 103.32)* | |
| Xu 2016 | 2011.10-2014.4 | China | UACR | Case-control I (DM) | 86 | 48 | 38 | 44.41±10.81 | 25.39±3.79 | 10.45±2.93 | NA | NA |
| | | | | Case-control II (DN) | 74 | 48 | 26 | 49.22±12.71 | 25.35±2.94 | 10.83±2.64 | NA | |
| Ao 2017 | 2014-2016 | China | eGFR | Case-control I (RFD) | 100 | 56 | 44 | 70.6±7.6 | 25.28±6.29 | NA | NA | NA |
| | | | | Case-control II (DN) | 100 | 55 | 45 | 56.4±8.5 | 26.99±3.34 | NA | NA | |
| Cao 2020 | 2018.10-2019.6 | China | UACR | Case-control I (DN) | 14 | 14 | 0 | 46.57±9.75 | 24.67±1.97 | NA | 60.27±7.30 | NA |
| | | | | Case-control III (RFD) | 59 | 36 | 23 | 56.14±9.57 | 26.13±3.37 | NA | 67.96±21.42 | |
| Chen 2024 | 2018.1-2023.6 | China | eGFR | Case-control | 100 | 68 | 32 | 56.06±2.11 | 22.73±1.04 | NA | NA | NA |
| Chen 2023 | 2017.5-2021.3 | China | eGFR | Case-control I (RFD) | 52 | 31 | 21 | 63.14±10.23 | NA | NA | 56.24±14.93 | 3.12 |
| | | | | Case-control II (DN) | 69 | 44 | 25 | 62.11±11.09 | NA | NA | 88.86±18.37 | |
| Fan 2022 | 2020.11 | China | NA | Case-control | 53 | 23 | 30 | 52.67±14.8 | NA | NA | NA | 4.01 |
| Li 2024 | 1999-2018 | American | DEATH | Case-control I (DN) | 1122 | 583 | 539 | 67.00 (60.00, 74.00)* | 31.60 (27.88, | 7.11 (5.61, 9.48)* | 48.17 (35.88, 56.20)* | NA |

| | | | | | | | | | | | | |
|---|---|---|---|---|---|---|---|---|---|---|---|---|
| | | | | | | | | | 36.61)* | | | |
| | | | | Case-control II (DEATH) | 921 | 424 | 497 | 74.00 (66.00, 80.00)* | 29.64 (26.84, 34.74)* | 7.27 (5.72, 10.10)* | 40.44 (27.37, 50.08)* | |
| Luo 2022 | 2015.4-2020.10 | China | eGFR | Case-control I (RFD) | 53 | 28 | 25 | 60.17±8.32 | NA | 8.37+1.12 | 45.44±9.48 | 3.5 |
| | | | | Case-control II (DN) | 44 | 27 | 17 | 61.91±8.13 | NA | 8.03±1.16 | 71.03±7.08 | |
| Shao 2022 | 2018.1-2021.4 | China | Scr | Case-control I (DN) | 41 | 30 | 10 | 52.98±10.86 | NA | 7.84 (5.38, 8.70)* | 117.18 (93.65, 156.45)* | |
| | | | | Case-control II (RFD1) | 40 | 21 | 19 | 51.90±9.87 | NA | 7.63 (5.32, 9.87)* | 86.84 (62.79, 121.25)* | 3.6 |
| | | | | Case-control III (RFD2) | 45 | 32 | 13 | 51.40±9.26 | NA | 7.42 (5.70, 8.13)* | 39.12 (28.09, 60.91)* | |
| Su 2024 | 2021.2-2024.2 | China | UAER | Case-control I (DN) | 49 | 46 | 34 | 62.49±4.06 | 23.38±1.50 | NA | 82.40±7.26 | NA |
| | | | | Case-control II (RFD) | 31 | NA | NA | NA | NA | NA | 61.32±6.09 | |
| Sun 2023 | 2019.1-2021.12 | China | UACR | Case-control | 125 | 99 | 26 | 52.84±10.70 | 25.44±3.33 | 7.28±3.68 | 52.45 (23.15, 80.89)* | 2.63 |
| Wang 2022 | 2021.10-2022.6 | China | UACR | Case-control I (DN) | 45 | 25 | 20 | 58.00±10.75 | 24.78±3.17 | 7.32±2.21 | 101.38 (94.01, 118.94)* | 1.925 |
| | | | | Case-control II (RFD) | 45 | 25 | 20 | 56.44±14.38 | 24.89±5.20 | 7.72±3.87 | 81.72 (72.66, 96.33)* | |
| Zhang 2019 | 2012.7-2018.6 | China | UAER | Case-control I (DN) | 30 | 16 | 14 | 57.53±9.61 | 24.73 (23.81, 27.44)* | 8.66±2.75 | 143.32±33.22 | 2.29 |
| | | | | Case-control II (RFD1) | 29 | 18 | 11 | 63.41±10.94 | 27.13 (23.77, | 8.01±3.63 | 36.01±9.78 | |

| | | | | | | | |
|---|---|---|---|---|---|---|---|
| | | | | | 28.66)* | | |
| Case-control III (RFD2) | 31 | 22 | 9 | 62.32±8.18 | 23.94 (20.50, 26.94)* | 10.70±4.41 | 6.71±4.77 |

Abbreviation: NLR, neutrophil-to-lymphocyte ratio; BMI, body mass index; FPG, fasting plasma glucose; RFD, renal function deterioration; eGFR, estimated glomerular filtration rate; UACR, urinary albumin-to-creatinine ratio; UAC, urinary albumin concentration; UAER, urinary albumin excretion rate; UAE, urinary albumin excretion; UPCR, urinary protein-to-creatinine ratio; NA, not available; #, median±range; *, median±interquartile range; NA, not available.

**Supplementary Table S2**. Quality evaluation of the eligible studies with Newcastle–Ottawa scale.

| Study | Selection | | | | Comparability | | Outcome | | | NOS | Study | Confounders adjusted |
|---|---|---|---|---|---|---|---|---|---|---|---|---|
| | S1 | S2 | S3 | S4 | C1 | C2 | O1 | O2 | O3 | scores | quality | |
| Chen 2022 | * | * | * | * | - | * | * | * | * | 8 | high | age, gender |
| Olores 2023 | * | * | * | * | - | * | * | * | * | 8 | high | age, gender, BMI, blood pressure |
| Li 2022 | * | * | * | * | - | - | * | * | * | 7 | high | age, gender, BMI, blood pressure |
| Kawamoto 2018 | * | * | * | * | - | * | * | * | * | 8 | high | age, gender, BMI, blood pressure |
| Suvarna1 2023 | * | * | * | * | - | * | * | * | * | 8 | high | -- |
| Wan 2020 | * | * | * | * | - | - | * | * | * | 7 | high | age, gender, BMI, blood pressure, HbA1c, duration of diabetes |
| Liu 2023 | * | * | * | * | - | * | * | * | * | 8 | high | gender, BMI |
| Tutan 2023 | * | * | * | * | - | * | * | * | * | 8 | high | gender, HbA1c, FPG |
| Zhang 2019 | * | * | * | * | - | - | * | * | * | 7 | high | age, gender, blood pressure, HbA1c |
| Kamrul-Hasan 2020 | * | * | * | * | - | - | * | * | * | 7 | high | gender, BMI, blood pressure, duration of diabetes |
| Chollang 2023 | * | * | * | * | - | * | * | * | * | 8 | high | age, gender, HbA1c |
| Mattared 2019 | * | * | * | * | - | * | * | * | * | 8 | high | age |
| Ciray 2015 | * | * | * | * | - | * | * | * | - | 7 | high | age, HbA1c, duration of diabetes, eGFR |
| Singh 2020 | * | * | * | * | - | * | * | * | * | 8 | high | age, gender, HbA1c, duration of diabetes |
| Jaaban 2021 | * | * | * | * | - | * | * | * | * | 8 | high | gender, BMI |
| Gurmu 2022 | - | - | * | * | - | * | * | * | * | 6 | moderate | gender, BMI, FPG |
| Huang 2014 | * | * | * | * | - | * | * | * | * | 8 | high | age, gender, BMI |
| Assulyn 2020 | * | * | * | * | - | * | * | * | * | 8 | high | BMI |
| Azab 2012 | * | * | * | * | - | * | * | * | * | 8 | high | blood pressure |
| Huang 2017 | * | * | * | * | - | - | * | * | * | 7 | high | age, gender, BMI, blood pressure, HbA1c, UACR |
| Akase 2020 | * | * | * | * | - | * | * | * | * | 8 | high | age, gender, BMI, blood pressure, duration of diabetes, UACR |
| Bloch 2020 | * | * | * | * | - | * | * | * | * | 8 | high | age, gender, HbA1c |

| | | | | | | | | | | | | |
|---|---|---|---|---|---|---|---|---|---|---|---|---|
| Subramani 2023 | * | * | * | * | - | * | * | * | * | 8 | high | gender, BMI |
| Khandare 2017 | * | * | * | * | - | * | * | * | * | 8 | high | age, gender, BMI, HbA1c, FPG |
| Gupta 2018 | * | * | * | * | - | * | * | * | * | 8 | high | age, BMI, HbA1c |
| Ge 2023 | * | * | * | * | - | * | * | * | * | 8 | high | age, gender, HbA1c, FPG |
| Xu 2016 | * | * | * | * | - | * | * | * | * | 8 | high | gender, BMI, blood pressure, Scr |
| Aao 2017 | * | * | * | * | - | * | * | * | * | 8 | high | age, gender, BMI, blood pressure, HbA1c, duration of diabetes, Scr |
| Cao 2020 | * | * | * | * | - | * | * | * | * | 8 | high | gender, BMI, blood pressure, HbA1c |
| Chen 2024 | * | * | * | * | - | * | * | * | * | 8 | high | age, gender, BMI |
| Chen 2023 | * | * | * | * | - | * | * | * | * | 8 | high | age, gender, blood pressure, HbA1c, duration of diabetes, FPG, Scr |
| Fan 2022 | * | * | * | * | - | * | * | * | * | 8 | high | age, gender, duration of diabetes |
| Li 2024 | * | * | * | * | - | - | * | * | * | 7 | high | age, gender, BMI, blood pressure, HbA1c, FPG, eGFR, Scr |
| Luo 2022 | * | * | * | * | - | * | * | * | * | 8 | high | age, gender, blood pressure, HbA1c, duration of diabetes, FPG |
| Shao 2022 | * | * | * | * | - | * | * | * | * | 8 | high | age, gender |
| Su 2024 | * | * | * | * | - | * | * | * | * | 8 | high | age, gender, BMI, duration of diabetes |
| Sun 2023 | * | * | * | * | - | - | * | * | * | 7 | high | age, gender, BMI, duration of diabetes |
| Wang 2022 | * | * | * | * | - | * | * | * | * | 8 | high | age, gender, BMI, blood pressure, HbA1c, FPG |
| Zhang 2019 | * | * | * | * | - | - | * | * | * | 7 | high | age |

"*" indicates that the criterion was met; "-" indicates that the criterion was not met.

S1, representativeness; S2, selection of non-exposed; S3, ascertainment of exposure; S4, outcome not present at start; C1, comparability on main factors; C2, comparability on other factors; O1, assessment of outcome; O2, follow-up ≥6month; O3, adequacy of follow-up.

NOS scores, Newcastle-Ottawa scale scores; BMI, body mass index; HbA1c, hemoglobin A1c; FPG, fasting plasma glucose; eGFR, estimated glomerular filtration rate; UACR, urine albumin-to-creatinine ratio; Scr, serum creatinine.

**Supplementary Table S3.** Studies contributing to heterogeneity and results after removing the outlier studies

| 1. NLR (continuous) vs. DN risk | | | | |
|---|---|---|---|---|
| | Studies contributing to heterogeneity | Results after removing the outlier studies | | |
| | | SMD [95%CI] | *P* value | $I^2$ |
| Overall | Assulyn 2020 B, Chen 2022, Gupta 2018 B, Gurmu 2022, Huang 2014, Jaaban 2021 B, Kamrul-Hasan 2020, Liu 2023 B, Subramani 2023 B | 1.08 [0.94, 1.21] | < 0.00001 | 36% |
| Region | | | | |
| South Asia | Gupta 2018 B, Gupta 2018 B, Kamrul-Hasan 2020 | 1.22 [1.06, 1.39] | < 0.00001 | 0% |
| East Asia | Chen 2022, Huang 2014, Li 2022, Liu 2023 B | 0.68 [0.38, 0.99] | < 0.0001 | NA |
| west Asia | Assulyn 2020 B, Jaaban 2021 B | 0.90 [0.53, 1.27] | < 0.00001 | 44% |
| Age, years | | | | |
| ≥ 60 | Assulyn 2020 B, Chollangi 2023 | 1.04 [0.77, 1.32] | < 0.00001 | NA |
| < 60 | Gurmu 2022, Gupta 2018 B, Subramani 2023 B, Jaaban 2021 B, Liu 2023 B, Liu 2023 A, Chen 2022, Huang 2014, Kamrul-Hasan 2020 | 1.19 [1.03, 1.34] | < 0.00001 | 0% |

| | | | | |
|---|---|---|---|---|
| NLR cut-off | | | | |
| ≥ 2.4 | Assulyn 2020 B, Li 2022 | 0.95 [0.57, 1.33] | < 0.00001 | NA |
| < 2.4 | Subramani 2023 B, Jaaban 2021 B, Chen 2022 | 1.15 [0.91, 1.38] | < 0.00001 | 0% |
| BMI, kg/m² | | | | |
| ≥ 25 | Subramani 2023 B, Jaaban 2021 B, Assulyn 2020 B, Kamrul-Hasan 2020 | 1.07 [0.94, 1.20] | < 0.00001 | 0% |
| < 25 | Gurmu 2022, Gupta 2018 B, Liu 2023 B, Liu 2023 A, Huang 2014 | 1.30 [1.02, 1.59] | < 0.00001 | NA |
| FPG, mmol/L | | | | |
| ≥ 9 | Gurmu 2022, Kamrul-Hasan 2020 | 1.18 [0.94, 1.43] | < 0.00001 | 0% |
| < 9 | Liu 2023 A, Liu 2023 B | 1.11 [0.95, 1.28] | < 0.00001 | NA |

NLR, neutrophil-to-lymphocyte ratio; DN, diabetic nephropathy; SMD, standardized mean difference; CI, confidence interval; BMI, body mass index; FPG, fasting plasma glucose; NA, not available.

| 2. NLR (continuous) vs. deterioration in renal function | | | | |
|---|---|---|---|---|
| | Studies contributed to heterogeneity | Results after removing the outlier studies | | |
| | | SMD [95%CI] | *P* value | $I^2$ |
| Overall | Su 2024, Li 2022, Cao 2020 | 1.05 [0.88, 1.22] | < 0.00001 | 39% |
| Age, years | | | | |
| ≥ 60 | Su 2024, Zhang 2019b, Li 2022 | 0.87 [0.65, 1.08] | < 0.00001 | 0% |
| < 60 | Cao 2020 | 1.08 [0.88, 1.29] | < 0.00001 | 38% |
| BMI, kg/m² | | | | |
| ≥ 25 | Subramani 2023, Jaaban 2021, Cao 2020 | 0.65 [0.43, 0.86] | < 0.00001 | 34% |
| < 25 | Su 2024 | 0.98 [0.76, 1.20] | < 0.00001 | 26% |
| Diagnostic markers | | | | |
| eGFR | NA | 0.83 [0.65, 1.01] | < 0.00001 | 0% |
| UACR | Subramani 2023, Jaaban 2021, Cao 2020 | 0.70 [0.49, 0.92] | < 0.00001 | 32% |

| | | | | |
|---|---|---|---|---|
| others | NA | 1.28 [1.03, 1.53] | < 0.00001 | 0% |

NLR, neutrophil-to-lymphocyte ratio; SMD, standardized mean difference; CI, confience interval; BMI, body mass index; FPG, fasting plasma glucose; eGFR, estimated glomerular filtration rate; UACR, urinary albumin-to-creatinine ratio; NA, not available.

**Supplementary Table S4.** PRISMA_2020_checklist

| Section and Topic | Item # | Checklist item | Location where item is reported (page) |
|---|---|---|---|
| **TITLE** | | | |
| Title | 1 | Identify the report as a systematic review. | 1 |
| **ABSTRACT** | | | |
| Abstract | 2 | See the PRISMA 2020 for Abstracts checklist. | 1-2 |
| **INTRODUCTION** | | | |
| Rationale | 3 | Describe the rationale for the review in the context of existing knowledge. | 2-3 |
| Objectives | 4 | **Provide an explicit statement of the objective(s) or question(s) the review addresses.** | 3 |
| **METHODS** | | | |
| Eligibility criteria | 5 | Specify the inclusion and exclusion criteria for the review and how studies were grouped for the syntheses. | 3-4 |
| Information sources | 6 | Specify all databases, registers, websites, organisations, reference lists and other sources searched or consulted to identify studies. Specify the date when each source was last searched or consulted. | 3 |
| Search strategy | 7 | Present the full search strategies for all databases, registers and websites, including any filters and limits used. | 3 |
| Selection process | 8 | Specify the methods used to decide whether a study met the inclusion criteria of the review, including how many reviewers screened each record and each report retrieved, whether they worked independently, and if applicable, details of automation tools used in the process. | 4 |
| Data collection process | 9 | Specify the methods used to collect data from reports, including how many reviewers collected data from each report, whether they worked independently, any processes for obtaining or confirming data from study investigators, and if applicable, details of automation tools used in the process. | 4 |
| Data items | 10a | List and define all outcomes for which data were sought. Specify whether all results that were compatible with each outcome domain in each study were | 4 |

| Section and Topic | Item # | Checklist item | Location where item is reported (page) |
|---|---|---|---|
| | | sought (e.g. for all measures, time points, analyses), and if not, the methods used to decide which results to collect. | |
| | 10b | List and define all other variables for which data were sought (e.g. participant and intervention characteristics, funding sources). Describe any assumptions made about any missing or unclear information. | 4 |
| Study risk of bias assessment | 11 | Specify the methods used to assess risk of bias in the included studies, including details of the tool(s) used, how many reviewers assessed each study and whether they worked independently, and if applicable, details of automation tools used in the process. | 4 |
| Effect measures | 12 | Specify for each outcome the effect measure(s) (e.g. risk ratio, mean difference) used in the synthesis or presentation of results. | 4 |
| Synthesis methods | 13a | Describe the processes used to decide which studies were eligible for each synthesis (e.g. tabulating the study intervention characteristics and comparing against the planned groups for each synthesis (item #5)). | 5, Supplementary Table S2 |
| | 13b | Describe any methods required to prepare the data for presentation or synthesis, such as handling of missing summary statistics, or data conversions. | 4 |
| | 13c | Describe any methods used to tabulate or visually display results of individual studies and syntheses. | 4 |
| | 13d | Describe any methods used to synthesize results and provide a rationale for the choice(s). If meta-analysis was performed, describe the model(s), method(s) to identify the presence and extent of statistical heterogeneity, and software package(s) used. | 4 |
| | 13e | Describe any methods used to explore possible causes of heterogeneity among study results (e.g. subgroup analysis, meta-regression). | 4 |
| | 13f | Describe any sensitivity analyses conducted to assess robustness of the synthesized results. | 4 |
| Reporting bias assessment | 14 | Describe any methods used to assess risk of bias due to missing results in a synthesis (arising from reporting biases). | 4 |
| Certainty assessment | 15 | Describe any methods used to assess certainty (or confidence) in the body of evidence for an outcome. | 4 |

| Section and Topic | Item # | Checklist item | Location where item is reported (page) |
|---|---|---|---|
| **RESULTS** | | | |
| Study selection | 16a | Describe the results of the search and selection process, from the number of records identified in the search to the number of studies included in the review, ideally using a flow diagram. | 5, Figure 1 |
| | 16b | Cite studies that might appear to meet the inclusion criteria, but which were excluded, and explain why they were excluded. | 5 |
| Study characteristics | 17 | Cite each included study and present its characteristics. | 5, Supplementary Table S1 |
| Risk of bias in studies | 18 | Present assessments of risk of bias for each included study. | 7 |
| Results of individual studies | 19 | For all outcomes, present, for each study: (a) summary statistics for each group (where appropriate) and (b) an effect estimate and its precision (e.g. confidence/credible interval), ideally using structured tables or plots. | 5-6, Table 1-3 |
| Results of syntheses | 20a | For each synthesis, briefly summarise the characteristics and risk of bias among contributing studies. | 5-6 |
| | 20b | Present results of all statistical syntheses conducted. If meta-analysis was done, present for each the summary estimate and its precision (e.g. confidence/credible interval) and measures of statistical heterogeneity. If comparing groups, describe the direction of the effect. | 5-6 |
| | 20c | Present results of all investigations of possible causes of heterogeneity among study results. | 7 |
| | 20d | Present results of all sensitivity analyses conducted to assess the robustness of the synthesized results. | 7, Figure 5 |
| Reporting biases | 21 | Present assessments of risk of bias due to missing results (arising from reporting biases) for each synthesis assessed. | 7 |
| Certainty of evidence | 22 | Present assessments of certainty (or confidence) in the body of evidence for each outcome assessed. | 7 |

| Section and Topic | Item # | Checklist item | Location where item is reported (page) |
|---|---|---|---|
| **DISCUSSION** | | | |
| Discussion | 23a | Provide a general interpretation of the results in the context of other evidence. | 7-8 |
| | 23b | Discuss any limitations of the evidence included in the review. | 10 |
| | 23c | Discuss any limitations of the review processes used. | 10 |
| | 23d | Discuss implications of the results for practice, policy, and future research. | 10 |
| **OTHER INFORMATION** | | | |
| Registration and protocol | 24a | Provide registration information for the review, including register name and registration number, or state that the review was not registered. | 3 |
| | 24b | Indicate where the review protocol can be accessed, or state that a protocol was not prepared. | 3 |
| | 24c | Describe and explain any amendments to information provided at registration or in the protocol. | 3 |
| Support | 25 | Describe sources of financial or non-financial support for the review, and the role of the funders or sponsors in the review. | 10 |
| Competing interests | 26 | Declare any competing interests of review authors. | 1 |
| Availability of data, code and other materials | 27 | Report which of the following are publicly available and where they can be found: template data collection forms; data extracted from included studies; data used for all analyses; analytic code; any other materials used in the review. | 10 |

**Supplementary Figure S1.** PRISMA_2020_flow diagram

**PRISMA 2020 flow diagram for new systematic reviews which included searches of databases and registers only**

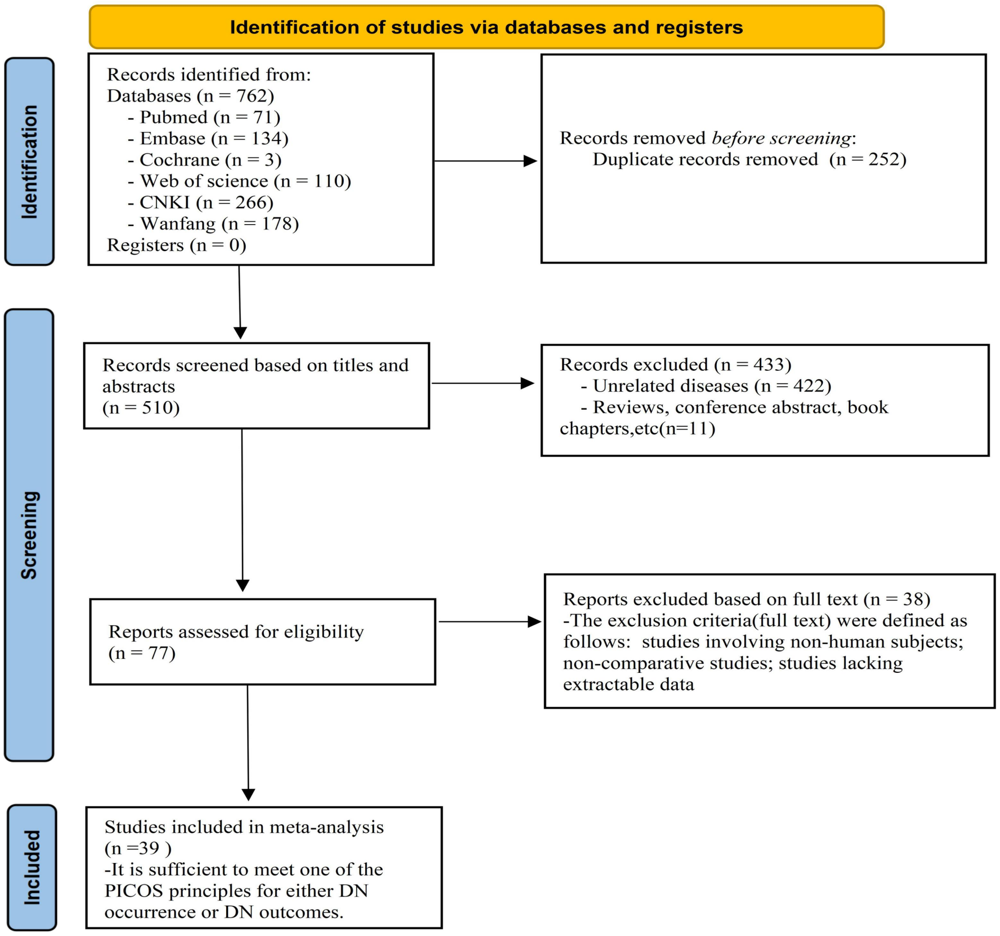


Source: Page MJ, et al. BMJ 2021;372:n71. doi: 10.1136/bmj.n71.

This work is licensed under CC BY 4.0. To view a copy of this license, visit https://creativecommons.org/licenses/by/4.0/

**Supplementary References.** Reference section with over 60 articles


61 Bloch MH, Iqbal F, Shafiq N, Bhatti A. Role of Neutrophil / Lymphocyte Ratio in Diabetes 2 Nephropathy. *Med Forum* **31**, 29-32 (2020).

62 Chen, X., Wang, Q. & Li, C. A retrospective analysis of hematologic parameters in patients with early diabetic kidney disease. *Clinical and Applied Thrombosis/Hemostasis* **28**, 10760296221083681 (2022).

63 Gupta, N., Karoli, R., Singh, P. S. & Shrivastava, A. The relationship between neutrophil/lymphocyte ratio, albuminuria and renal dysfunction in diabetic nephropathy. *J Indian Acad Clin Med* **19**, 265-268 (2018).

64 XU, X., ZHONG, X. & PAN, T. Relationship between neutrophil-to-lymphocyte ratio and early-stage diabetic nephropathy in patients with; newly diagnosed type 2 diabetes. *Chinese Journal of Diabetes*, 598-600 (2016).

65 Akase, T., Kawamoto, R., Ninomiya, D., Kikuchi, A. & Kumagi, T. Neutrophil-to-lymphocyte ratio is a predictor of renal dysfunction in Japanese patients with type 2 diabetes. *Diabetes & Metabolic Syndrome: Clinical Research & Reviews* **14**, 481-487 (2020).

66 Huang, L., Xie, Y., Dai, S. & Zheng, H. Neutrophil-to-lymphocyte ratio in diabetic microangiopathy. *Int J Clin Exp Pathol* **10**, 1223-1232 (2017).

67 Assulyn, T., Khamisy-Farah, R., Nseir, W., Bashkin, A. & Farah, R. Neutrophil-to-lymphocyte ratio and red blood cell distribution width as predictors of microalbuminuria in type 2 diabetes. *Journal of Clinical Laboratory Analysis* **34**, e23259 (2020).

68 Huang, W. *et al.* Neutrophil–lymphocyte ratio is a reliable predictive marker for early-stage diabetic nephropathy. *Clinical endocrinology* **82**, 229-233 (2015).

69 Jaaban, M., Zetoune, A. B., Hesenow, S. & Hessenow, R. Neutrophil-lymphocyte ratio and platelet-lymphocyte ratio as novel risk markers for diabetic nephropathy in patients with type 2 diabetes. *Heliyon* **7** (2021).

70 Singh, A., Jha, A. K., Kalita, B. C., Jha, D. K. & Alok, Y. Neutrophil lymphocyte ratio: a reliable biomarker for diabetic nephropathy? *International Journal of Diabetes in Developing Countries* **42**, 523-528 (2022).

71 Chollangi, S. *et al.* Exploring the correlates of hematological parameters with early diabetic nephropathy in type 2 diabetes mellitus. *Cureus* **15** (2023).

72 Tutan, D. & Doğan, M. Evaluation of neutrophil/lymphocyte ratio, low-density lipoprotein/albumin ratio, and red cell distribution width/albumin ratio in the estimation of proteinuria in uncontrolled diabetic patients. *Cureus* **15** (2023).

73 Kamrul-Hasan, A. *et al.* Evaluation of neutrophil–lymphocyte ratio and platelet–lymphocyte ratio as markers of diabetic kidney disease in Bangladeshi patients with type 2 diabetes mellitus. *Journal of Diabetology* **12**, 58-62 (2021).

74 Zhang, J. *et al.* Effects of neutrophil–lymphocyte ratio on renal function and histologic lesions in patients with diabetic nephropathy. *Nephrology* **24**, 1115-1121 (2019).

75 Ge D, C. Z., Si HF, Ling HF. Correlation between Hypersensitive C - Reactive Protein / Albumin Ratio and Diabetic Kidney Disease. *Chin J Diabetes* **31**, 413-417, doi:10. 3969/j. issn. 1006- 6187. 2023. 06. 003 (2023).

76 Kawamoto, R. *et al.* Association of neutrophil-to-lymphocyte ratio with early renal dysfunction and albuminuria among diabetic patients. *International urology and nephrology* **51**, 483-490 (2019).

77 Suvarna, R., Biswas, M., Shenoy, R. P. & Prabhu, M. M. Association of clinical variables as a predictor marker in type 2 diabetes mellitus and diabetic complications. *Biomedicine* **43**, 335-340 (2023).

78 Olores, L. A., Darunday, G., Polito, E. & Maguad, R. Association between Neutrophil-Lymphocyte Ratio (NLR) and Clinical Outcomes among Filipino Patients with End-Stage Renal Disease (ESRD) Secondary to Diabetic Nephropathy on Maintenance Hemodialysis. *Open Access Library Journal* **10**, 1-10 (2023).

79 Ito, S. *et al.* Neutrophil/lymphocyte ratio elevation in renal dysfunction is caused by distortion of leukocyte hematopoiesis in bone marrow. *Renal Failure* **41**, 284-293 (2019).

80 Navarro-Gonzalez, J. F. & Mora-Fernandez, C. The role of inflammatory cytokines in diabetic nephropathy. *Journal of the American Society of Nephrology* **19**, 433-442 (2008).

81 Chow, F. Y., Nikolic-Paterson, D. J., Atkins, R. C. & Tesch, G. H. Macrophages in streptozotocin-induced diabetic nephropathy: potential role in renal fibrosis. *Nephrology Dialysis Transplantation* **19**, 2987-2996 (2004).

82 Tang, S. C. & Lai, K. N. The pathogenic role of the renal proximal tubular cell in diabetic nephropathy. *Nephrology Dialysis Transplantation* **27**, 3049-3056 (2012).

83 Ha, H. & Lee, H. B. Reactive oxygen species amplify glucose signalling in renal cells cultured under high glucose and in diabetic kidney. *Nephrology* **10**, S7-S10 (2005).

84 Bauernfeind, F. *et al.* Cutting edge: reactive oxygen species inhibitors block priming, but not activation, of the NLRP3 inflammasome. *The Journal of Immunology* **187**, 613-617 (2011).

85 Tesch, G. H. Macrophages and diabetic nephropathy. *Semin Nephrol* **30**, 290-301, doi:10.1016/j.semnephrol.2010.03.007 (2010).

86 Chollangi, S., Rout, N. K. & Patro, S. A Study on Correlation of Neutrophil to Lymphocyte Ratio and Red Cell Distribution Width with Microalbuminuria in Type 2 Diabetes Mellitus. *The Journal of the Association of Physicians of India* **70**, 11-12 (2022).

87 Ruster, C. & Wolf, G. The role of chemokines and chemokine receptors in diabetic nephropathy. *Front Biosci* **13**, 944-955 (2008).

88 Kim, H. Y. *et al.* Uremic toxin indoxyl sulfate induces trained immunity via the AhR-dependent arachidonic acid pathway in end-stage renal disease (ESRD). *Elife* **12**, RP87316 (2024).

89 Fard, R. M., Rashno, M., & Bahreiny, S. S. Effects of melatonin supplementation on markers of inflammation and oxidative stress in patients with diabetes: A systematic review and meta-analysis of randomized controlled trials. *Clinical Nutrition ESPEN* **63**, 530-539 (2024).

90 Fang, Y. *et al.* Sipeimine ameliorates osteoarthritis progression by suppression of NLRP3 inflammasome-mediated pyroptosis through inhibition of PI3K/AKT/NF-κB pathway: An in vitro and in vivo study. *Journal of Orthopaedic Translation* **46**, 1-17 (2024).

90 Bu, J. *et al.* Acacetin inhibits inflammation by blocking MAPK/NF-κB pathways and NLRP3 inflammasome activation. *Frontiers in Pharmacology* **15**, 1286546 (2024).

92 Loeffler, I. & Wolf, G. Transforming growth factor-β and the progression of renal disease. *Nephrology Dialysis Transplantation* **29**, i37-i45 (2014).

93 Turkmen, K., Guney, I., Yerlikaya, F. H. & Tonbul, H. Z. The relationship between neutrophil-to-lymphocyte ratio and inflammation in end-stage renal disease patients. *Renal failure* **34**, 155-159 (2012).

94 Bahreiny, S. S. *et al.* Integrative regression modeling of insulin sensitivity, resistance, and beta-cell dysfunction in predicting female infertility: a cross-sectional NHANES study. *Clinical*

*and Experimental Medicine* **25**, 1-18 (2025).
